# A novel iron bioresorbable scaffold: a potential strategy for pulmonary artery stenosis

**DOI:** 10.1093/rb/rbaf041

**Published:** 2025-05-13

**Authors:** Li Qin, Gui Zhang, Ling Sun, Zhijin Yu, Zhe Zhang, Lifeng Sun, Wanqian Zhang, Wenchao Fu, Yetao Ou, Wenjing Zhang, Xiaoli Shi, Zhixiang Si, Jingfang Shen, Limei Cha, Zhiwei Zhang, Deyuan Zhang

**Affiliations:** National and Local Joint Engineering Laboratory of Interventional Medical Biotechnology and System, Biotyx Medical (Shenzhen) Co., Ltd., Lifetech Scientific (Shenzhen) Co., Ltd., Shenzhen 518110, China; National and Local Joint Engineering Laboratory of Interventional Medical Biotechnology and System, Biotyx Medical (Shenzhen) Co., Ltd., Lifetech Scientific (Shenzhen) Co., Ltd., Shenzhen 518110, China; Department of Pediatric Cardiology, Guangdong Provincial People’s Hospital, Guangdong Academy of Medical Sciences, Guangdong Cardiovascular Institute, Guangzhou 510080, China; Materials Science and Engineering Department, Guangdong Technion-Israel Institute of Technology, Shantou 515063, China; Queen Mary School, Nanchang University, Nanchang 330038, China; Biotechnology and Food Engineering Department, Guangdong Technion-Israel Institute of Technology, Shantou 515063, China; National and Local Joint Engineering Laboratory of Interventional Medical Biotechnology and System, Biotyx Medical (Shenzhen) Co., Ltd., Lifetech Scientific (Shenzhen) Co., Ltd., Shenzhen 518110, China; National and Local Joint Engineering Laboratory of Interventional Medical Biotechnology and System, Biotyx Medical (Shenzhen) Co., Ltd., Lifetech Scientific (Shenzhen) Co., Ltd., Shenzhen 518110, China; National and Local Joint Engineering Laboratory of Interventional Medical Biotechnology and System, Biotyx Medical (Shenzhen) Co., Ltd., Lifetech Scientific (Shenzhen) Co., Ltd., Shenzhen 518110, China; Materials Science and Engineering Department, Guangdong Technion-Israel Institute of Technology, Shantou 515063, China; National and Local Joint Engineering Laboratory of Interventional Medical Biotechnology and System, Biotyx Medical (Shenzhen) Co., Ltd., Lifetech Scientific (Shenzhen) Co., Ltd., Shenzhen 518110, China; Materials Science and Engineering Department, Guangdong Technion-Israel Institute of Technology, Shantou 515063, China; Materials Science and Engineering Department, Guangdong Technion-Israel Institute of Technology, Shantou 515063, China; Materials Science and Engineering Department, Guangdong Technion-Israel Institute of Technology, Shantou 515063, China; Department of Pediatric Cardiology, Guangdong Provincial People’s Hospital, Guangdong Academy of Medical Sciences, Guangdong Cardiovascular Institute, Guangzhou 510080, China; National and Local Joint Engineering Laboratory of Interventional Medical Biotechnology and System, Biotyx Medical (Shenzhen) Co., Ltd., Lifetech Scientific (Shenzhen) Co., Ltd., Shenzhen 518110, China

**Keywords:** big and biodegradable scaffold, nitrided iron, pulmonary artery stenosis, congenital heart diseases, intervention

## Abstract

A big diameter bioresorbable scaffold is expected to be used for treatment of vessel stenosis of children with congenital heart disease to adapt the growth characteristics of vessel of children and avoid the late adverse events of permanent stent implanted in children. However, it is challenging to fabricate a big diameter bioresorbable scaffold that is appropriate for percutaneous implantation with enough mechanical performance and can be smoothly delivered in children’s small vessel. In this study, a novel iron big and bioresorbable Scaffold (BBS) for pulmonary artery stenosis of children with congenital cardiovascular diseases was fabricated and evaluated. The BBS was made of nitrided iron tube and processed by laser cutting and polishing. The testing results of radial strength, recoil, shortening, maximal expansion diameter and side-branch accessability illustrated the BBS has good mechanical performance. The animal study showed that the percentage of area stenosis of BBSs was 18.1 ± 8.6%, 20.2 ± 5.9% and 20.4 ± 6.1% at 28, 90 and 180 days after implantation in 17 rabbits, and no malposition, thrombus, dissection or tissue necrosis in the rabbit model was detected by micro-CT, STEM and histological examinations. An φ8 × 23 mm BBS was implanted into a 55-month-old child with left pulmonary stenosis, and multiple spiral CT was conducted. No lumen area loss appeared at 1- and 2-year follow-ups in this first-in-man study. It suggested that the BBS might be a new strategy for the therapy of pulmonary artery stenosis in children.

## Graphical abstract

**Figure:**
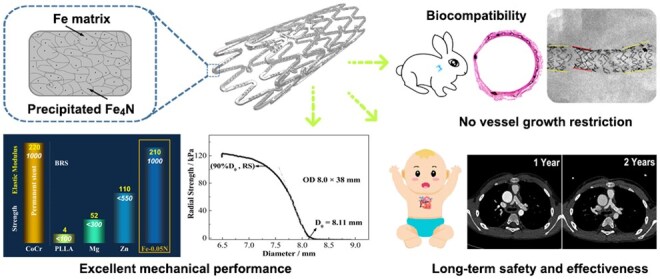

## Introduction

Pulmonary artery stenosis (PAS), including congenital and acquired, is commonly associated with congenital heart disease (CHD), such as the tetralogy of Fallot (TOF) [1–4]. Currently, the main treatment procedure for PAS is a surgery using patches to widen the pulmonary artery. However, surgical procedures probably pose a high risk to children with poor physical condition and still remain a significant challenge for distal pulmonary artery stenosis. In addition, surgery leads to significant vascular injury, and scar tissues come with the patched pulmonary arteries, causing acquired PAS. The surgical procedure is probably difficult since it is hard to separate the pulmonary artery from the scar tissue. The interventional procedure may solve this problem, such as pulmonary artery balloon angioplasty and stent implantation [5–7]. However, the results of balloon angioplasty are often not ideal, and the incidence of restenosis is high [8]. Therefore, pulmonary artery stent implantation may become a reasonable alternative to surgery in some cases. At the moment, all pulmonary artery stents in clinical use, including off-label-use stents and only one commercial pulmonary artery stent, are permanent ones. However, permanent stents have some limitations for children [9, 10]. Due to the non-degradable properties of the permanent stent, acquired PAS will occur when the children grow up. In this case, the children need to undergo a surgical operation to expand the acquired PAS, which may also encounter the difficulty of separating the blood vessels from the scar tissue. As an alternative method, implanting larger stents into the acquired PAS may solve this problem, however, the operation often fails because the current interventional techniques can hardly break the permanent scaffold longitudinally. Furthermore, the stent fracture of permanent stent used in PA stenting was reported [11], which may bring late safety problem.

Bioresorbable artery scaffold may solve the problems above [12–14]. After the vascular remodeling completes, the scaffold will gradually degrade and be absorbed by the body. Then, it will no longer restrict the growth and development of the children's blood vessels. If the scaffolded pulmonary artery grows too slowly to match the growth of the normal pulmonary artery and acquires restenosis, another bioresorbable scaffold can be implanted to widen the scaffolded pulmonary artery to accommodate the growth of children. Unfortunately, polymer or magnesium scaffold is too small for pulmonary intervention since the diameter of pulmonary artery varies between 4 and 24 mm from baby to adult. The preparation of large diameter bioabsorbable scaffold with mechanical properties that meets clinical requirements for children with congenital heart disease is a major challenge for polymer and magnesium scaffolds.

In this study, a novel iron big and bioresorbable scaffold (BBS, Biotyx Medical, previously developed at Lifetech Scientific Company) dedicated to treat PAS was successfully developed and evaluated. This novel iron bioresorbable scaffold was the first large diameter (>4.0 mm) metallic bioresorbable scaffold all over the world. The high-strength nitrided iron tube was designed to fabricate the BBS. The effects of N concentration on the tensile strength of the nitrided iron tube, as well as the radial strength of the BBS were discussed. The performance of device was also investigated. Using rabbit as the animal model, the degradation profile of the BBS *in vivo* was characterized. The histopathological observation was carried out to further investigate the local tissue response to the implanted BBS. First-in-Man implantation was also conducted to evaluate the safety and effectiveness of the BBS in the human body. The study showed that the BBS has good device performance, biocompatibility and effectiveness, making it a new treatment method for CHD patients with PAS.

## Materials and methods

### Materials

#### High-strength nitrided iron tube

The high-strength nitrided iron tube was prepared through the following steps. The pure iron tube (Fe ≥ 99.5 wt%) was nitrided by gas nitriding (NH_3_ ≥ 99.9 wt%) at 520°C for 30 min, then, annealing at 920°C for 30 min, ensuring a nitrided iron tube with uniform distribution of nitrogen (outer diameter (OD) 6 mm, wall thickness 0.5 mm) was obtained. The nitrided iron tube was further processed by repeating cold drawing and heat treatment to reduce the tube diameter and increase the tube strength. After straightening, a finished nitrided iron tube with 2.5/1.8 mm in OD was finally obtained. The tensile strength of the nitrided iron tube were tested on a short segment (∼10 cm) cut from the finished nitrided iron tube using universal tensile testing machine (C43.504, MTS SYSTEMS, USA). N concentration testing using was conducted by oxygen nitrogen hydrogen analyzer (ONH-2000, VERDER SCIENTIFIC, Germany) on the sample from the same tube.

#### Manufacture and characterization of BBS

**Figure 1.**
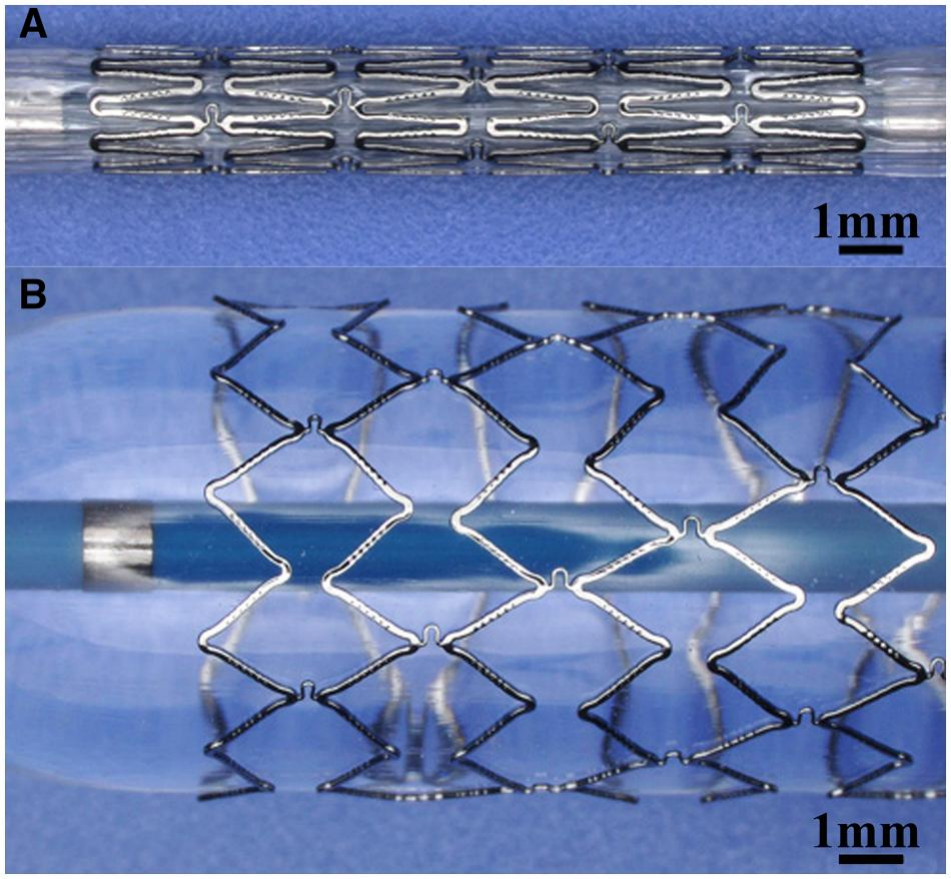
(**A**) The BBS mounted on the balloon. (**B**) After balloon inflation.

All BBSs (Figure 1) used in this study were manufactured in Biotyx Medical Co., Ltd Shenzhen, China. The BBSs were made of high-strength nitrided iron tube that was processed by laser cutting and chemical polishing, then, the BBSs were crimped onto a balloon catheter operated with an automatic crimping machine (Model CX, Blockwise, USA) and sterilized with ethylene oxide.

The composition and microstructure of the BBS (nitrided iron tube) were characterized by Scanning Transmission Electron Microscopy (STEM, Talos F200X, Thermo Scientific, USA) under an accelerating voltage of 200 kV. All STEM samples were prepared by Focused Ion Beam (FIB, Helios 5 UC, Thermo Scientific, USA).

### Device profile and its mechanical performance

#### Strut thickness, percent surface area and crossing profile

Strut thickness and percent surface area of the BBS were reported and calculated according to design drawings. The percent surface area of the BBS was calculated using Computer Aided Design (CAD) software from the design drawings. The percent surface area of the BBS was determined according to the following equation:

(1) $$\text{Percent}\;\text{surface}\;\text{area}\;\left(\%\right)=(S_{0}/S_{1})\times 100\%,$$

where S_0_ indicated the expanded scaffold at nominal pressure (NP) in contact with the vessel and S_1_ indicated full cylindrical surface area at the expanded scaffold diameter and length at NP.

The crossing profile of the BBS system was defined as the maximum diameter of the crimped scaffold and tested by digital microscope (VHX-970F, KEYENCE, Japan).

#### Foreshortening and recoil

Foreshortening and recoil were tested by digital microscope (VHX-970F, KEYENCE, Japan). The BBSs were crimped on balloon catheters in advance. Then scaffolds were deployed into mock vessels (inner diameter 7.2 ± 0.15 mm, radial compliance 5–7% per 100 mmHg@72 bpm, Dynatec Labs Inc., Galena, MO, USA) in PBS solution at 37 ± 2°C. The lengths of the BBS were measured both before balloon inflation and after the removal of the balloon catheter. The diameters of the BBS in the mock vessel were measured both with the inflated balloon inside under nominal pressure after 30 s dwell time and after the removal of the balloon catheter. The foreshortening and recoil of the BBS were determined according to Eqs. (2) and (3), respectively:

(2) $$\text{Foreshortening}\;\left(\%\right)=\left(1–\;L_{1}/L_{0}\right)\times 100\%,$$

where *L*_0_ indicated the original length of the BBS before inflation and *L*_1_ indicated the length of the BBS when the balloon was inflated to nominal pressure and then removed.

(3) $$\text{Recoil}\;\left(\%\right)=\left(1-D_{1}/D_{0}\right)\times 100\%,$$

where *D*_0_ indicated the outer diameter of the BBS while the balloon was inflated and D_1_ indicated the outer diameter of the BBS after the deflation of the balloon.

#### Radial strength and stiffness

The radial strength of the scaffold was tested using a radial force-tester method in which the scaffold was compressed circumferentially with a compression rate of 0.1 mm/s (RX550-100, Machine Solution Inc., USA). Radial strength (kPa) is defined as the strength when the outer diameter of the scaffold was compressed to 90% of its original value. The slope of the linear segment indicates scaffold radial stiffness (kPa/mm).

#### Side-branch accessability

Side-branch accessability was defined as the maximal inner diameter of the scaffold cell, to which the scaffold cell could maintain the integrity of scaffold structure with no strut fractures. A balloon was used to expand the closed cell of the scaffold formed by supporting wave rings and connecting units. A digital microscope (VHX-970F, KEYENCE, Japan) was employed to observe appearance and measure the diameter of the expanded cell.

Each of these experiments tested At least five samples.

### Implantation experiment

Seventeen BBSs (scaffold specification: φ 5.0 × 8 mm) were implanted into the pulmonary arteries of 17 rabbits (11 male rabbits and 6 female rabbits) purchased from Pearl Laboratory Animal Science & Technology Co., Ltd. The rabbits were fed with a standard diet throughout the experiment. The femoral vein was surgically exposed and a 5F introducer sheath was introduced over a 0.014 inch guidewire. Then, the BBS was introduced to the pulmonary artery along the femoral vein, inferior vena cava, right atrium, tricuspid valve and right ventricle. And finally, the scaffold arrived in the pulmonary artery of the rabbit. RX balloon catheters (φ 5.0 × 8 mm, Biotyx Medical, Shenzhen, China) were inflated under a pressure of 6 atm with a dwell time of 30 s to deploy the BBS. Then, the balloon catheter, 5F introducer sheath and guide wire were removed before the blood vessel was ligated.

All experimental animals used in the study were in accordance with accepted institutional policies, with the New Zealand rabbits under the approval of the Ethics Committee of Shenzhen Advanced Medical Services (AASB190501R).

#### In vivo degradation

At each follow-up time point, the rabbits were sacrificed and the scaffolded segments were explanted carefully. The BBSs with tissues were scanned through by micro-CT (Skyscan1172, Bruker, Germany) and 2D imaging was conducted to characterize the BBSs degradation. Then two scaffolded segments at each follow-up time point were prepared for histopathologic observation. The other three scaffolded segments were immersed in sodium hydroxide solution (1 mol/l) for 24 hr to dissolve tissue and ultrasonically cleaned in tartaric acid (5 wt%), sodium hydroxide solution (1 mol/l), deionized water and absolute ethyl alcohol in sequence to clean the degradation products, and then, weighed by an electronic balance (ME5, Sartorius, Germany) for calculating the mass loss of the scaffold. Another two explanted scaffolded segments at 180 days after implantation used to characterize the composition of the degradation products were immersed in sodium hydroxide solution (1 mol/l) for 24 hr to dissolve tissue, and then, the degradation products were peeled off from the scaffold struts, ultrasonically cleaned in absolute ethyl alcohol and dried at room temperature. The structure and composition of the degradation products were investigated by STEM (Talos F200X, Thermo Scientific, USA) under an accelerating voltage of 200 kV. All STEM samples were prepared by FIB (Helios 5 UC, Thermo Scientific, USA).

#### Histopathology

The explanted scaffolded pulmonary artery segments were fixed with 4% (w/v) paraformaldehyde, dehydrated and embedded them in resin. Cut the resin was cut into ∼ 150 μm thickness slices with the resin microtome (Lsomet 5000, BUEHLER, USA), and then polish the pieces into 10–20 μm slices with the grounding machine (Ecomet 250, BUEHLER, USA). The slices were stained by haematoxylin and eosin (HE). The pulmonary local-tissue response was observed afterward using a biological microscope (DM2500, Leica, Germany).

### First-in-man implantation

A 55-month-old child underwent percutaneous pulmonary intervention for diameter stenosis (∼50%) in the left pulmonary artery. The procedure was carried out at Guangdong Provincial People’s Hospital (Guangzhou, China). A φ 8 × 23 mm BBS was implanted in the left pulmonary via the right cardiac system and the right femoral vein approach. Pulmonary angiography was performed before and after implantation, multiple spiral CT imaging was performed at 1- and 2-year follow-up. The patient was required to receive 5 mg/kg of aspirin daily for 6 months and 1 mg/kg of clopidogrel daily for 1 month after implantation. The clinical study of the first-in-human implantation of the BBS was approved by the Ethics Committee of Guangdong Provincial People’s Hospital (2019-99, Guangzhou, China).

### Statistical methods

We used descriptive statistical methods to present the data. All results were expressed as mean ± standard deviation.

## Results

### High strength nitrided iron tube

**Figure 2.**
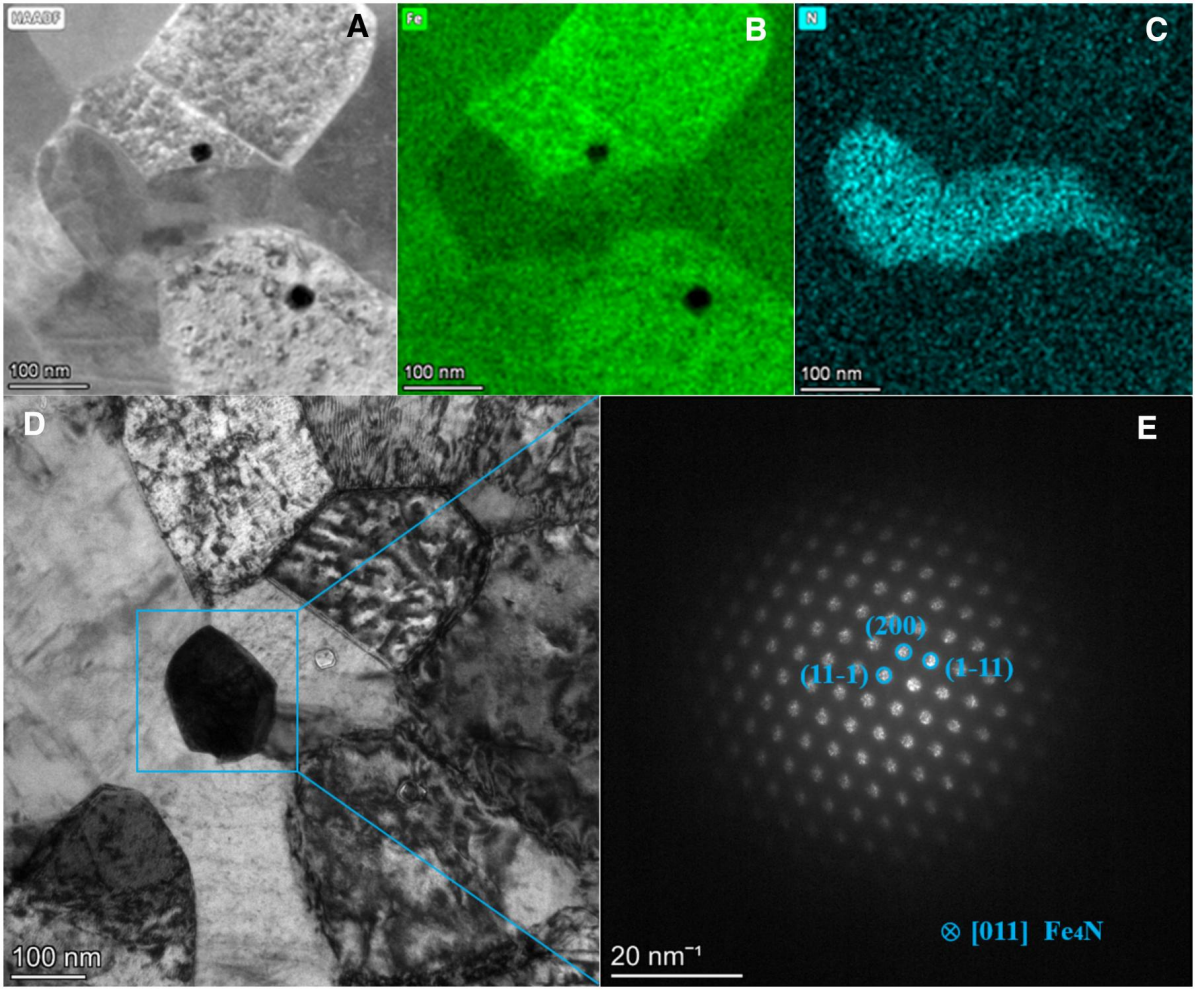
(**A**) HAADF-STEM image of the nitrided iron tube. (**B**) EDS mapping of Fe. (**C**) EDS mapping of N. (**D**) Bright field TEM images of a Fe_4_N grain from (**A**) viewed in the zone axis of [011]. (**E**) SAED pattern of the Fe_4_N grain in (**D**).

STEM was used to investigate the distribution of different phases and elements in the nitrided iron tube. A HAADF-STEM image is showed in Figure 2A, and related EDS mapping was performed (Figure 2B and C). The EDS mappings show that there is an N-rich area in the Fe matrix. A bright field image (Figure 2D) was taken from the same area, where the black grain is corresponding to the left part of the N-rich area in Figure 2A. It is on zone axis, and thus, shows a different contrast with the surrounding grains. The selected area electron diffraction (SAED) pattern (Figure 2E) indicates the black gain is Fe_4_N (JCPDS #no. 06-0627), where its (1$\overset{-}{1}$1), (11$\overset{-}{1}$) and (200) plane are viewed along the zone axis of [011].

**Figure 3.**
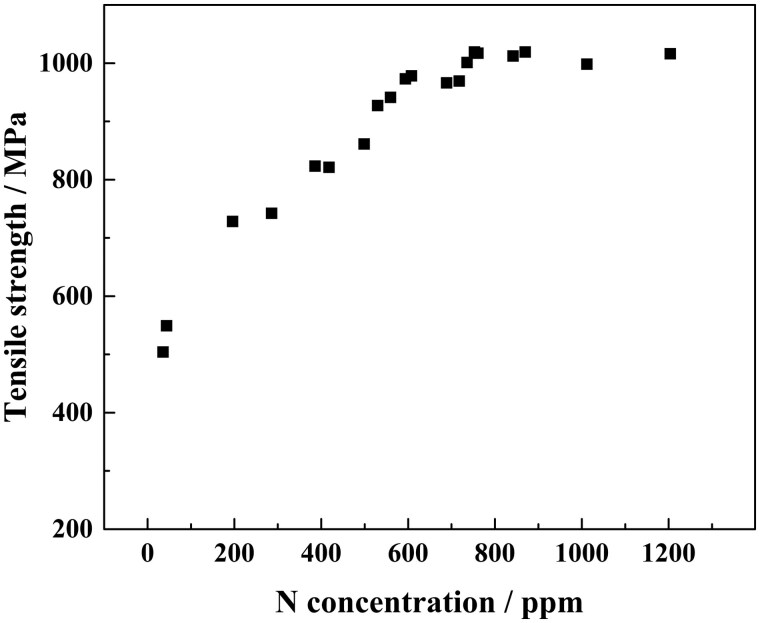
The relationships between the N concentration and the tensile strength of the nitrided iron tube.

The correlation between the tensile strength of the nitrided iron tube and N concentration in the nitrided iron tube are displayed in Figure 3, which shows that the tensile strength of the nitrided iron tube is approximately proportional to the N concentration in the range of 40 ppm to 700 ppm. The tensile strength almost keeps constant when the N concentration is higher than 700 ppm.

### The device performance

Table 1 shows the device parameters and performance of the BBS. The strut thickness of the BBS is only ∼105 μm. According to Eq. (1), the calculated percent surface area of the BBS is 10.6%, which is nearly the same as the permanent stent. The minimum sheath for delivering the BBS with a diameter of 8 mm and crimped on the over the wire balloon catheter is 6F. According to Eqs. (2) and (3), the calculated foreshortening and recoil of BBSs are 3.90 ± 0.09% and 0.35 ± 0.12%.

**Table 1.** Device parameters and performance of the BBS (φ 8.0 × 38 mm)

Strut thickness (μm)	Percent surface area @ NP(%)	Radial strength （kPa)	Radial stiffness (kPa/mm)	Minimum sheath （F）	Recoil @ NP (%)	Foreshortening @ NP (%)	Maximal expansion diameter (mm)	Side-branch accessability (mm)
105	10.6	100 ± 5	205 ± 15	6	3.90 ± 0.09	0.35 ± 0.12	11.5	5.0

**Figure 4.**
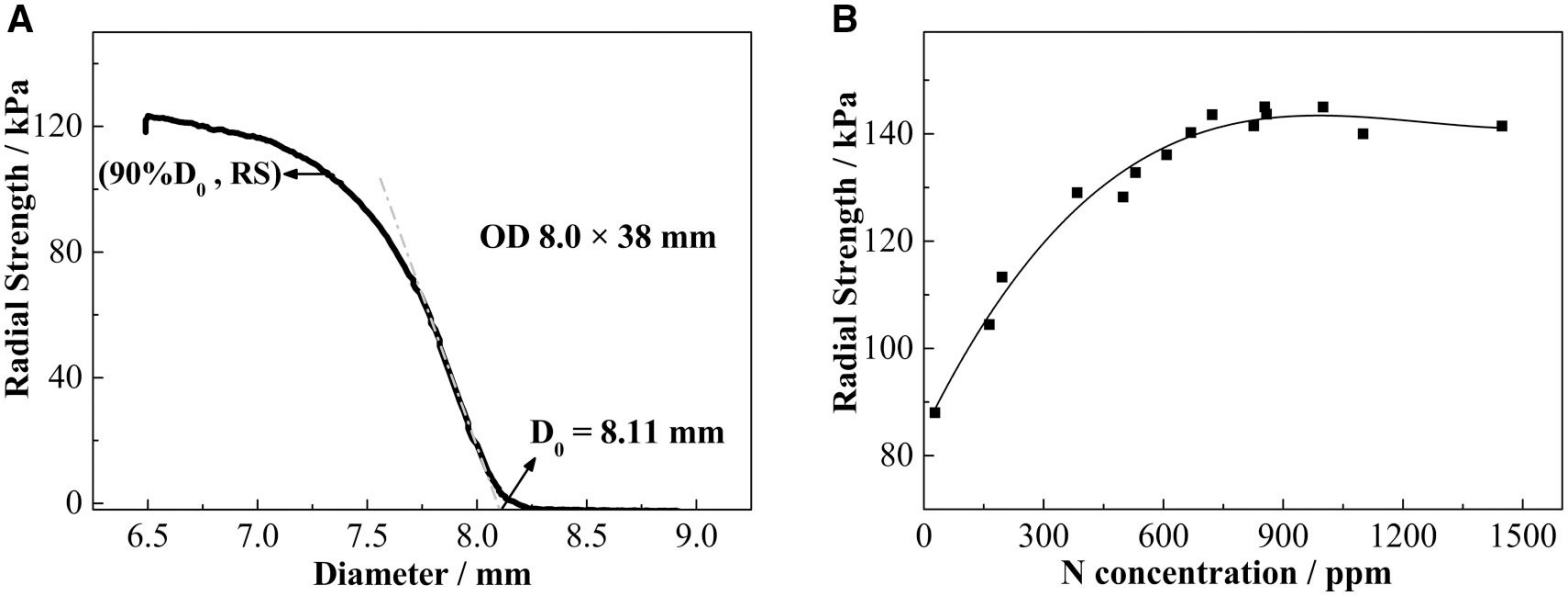
(**A**) Typical radial compression curve of the BBS. (**B**) The effects of the N concentration on the radial strength of the BBS.

Figure 4A is the typical radial compression curve (radial strength vs. scaffold diameter) of the BBS, where the linear segment indicates elastic deformations, and the slope of the linear segment is the scaffold radial stiffness (kPa/mm). The intersection of the linear segment and horizontal axis is defined as the original diameter *D*_0_ of the BBS @NP. The radial strength and radial stiffness of the BBS with 105 μm thickness are 100 ± 5 kPa and 205 ± 15 kPa/mm, respectively (listed in Table 1). The correlation between the N concentration and the radial strength of the BBSs (φ 4 × 38 mm) are shown in Figure 4B. It can be seen that the corresponding radial strength of BBS ranges from 85 kPa to 145 kPa, and the radial strength of the BBS is approximately proportional to N concentration when the N concentration increasing from 40 ppm to 700 ppm. When the N concentration of the scaffold is higher than 700 ppm, the radial strength almost keeps constant.

**Figure 5.**
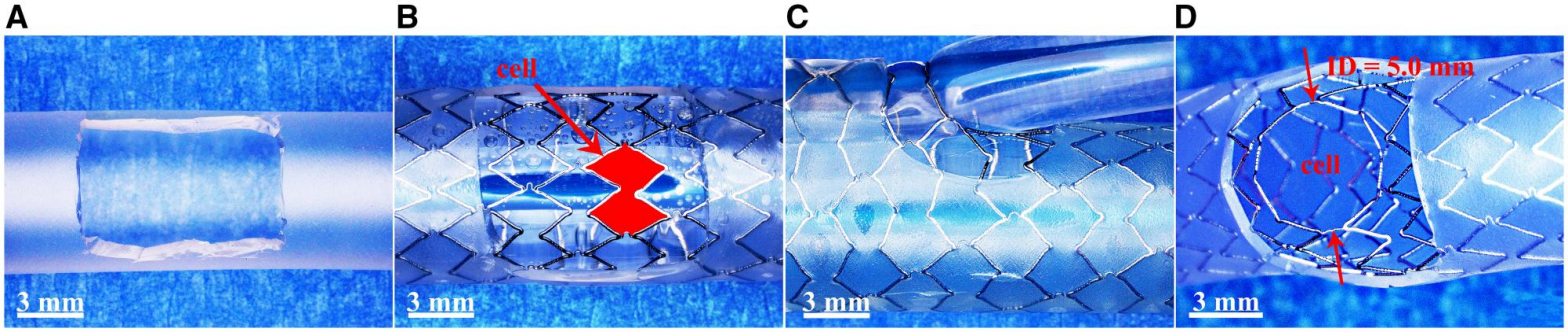
Evaluation of the side-branch accessability of a BBS with a size of φ 8.0 × 38 mm. (**A**) A fenestrated mock vessel, (**B**) the BBS was deployed with a cell at the window of the fenestrated mock vessel, (**C**) the cell was then dilated with a φ 8.0 mm balloon and (**D**) measuring of the inner diameter of a cell after dilation.

As shown in Figure 5, the maximal expansion diameter and side-branch accessability of the BBS with a size of φ 8.0 × 38 mm were tested, and the corresponding values are 11.5 mm and 5.0 mm, respectively (listed in Table 1). The cell of the scaffold is usually dilated to design limit in clinical application to ensure its side-branch accessability.

### Animal study

#### In vivo degradation profile

**Figure 6.**
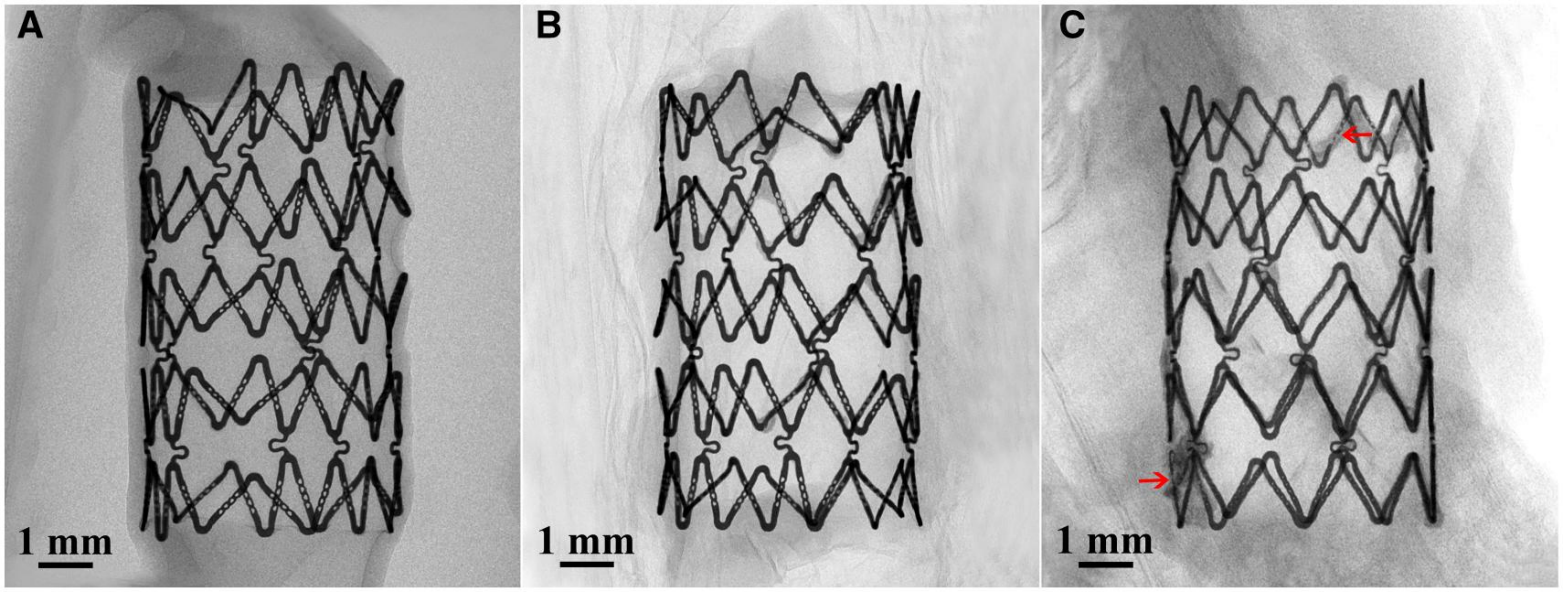
Typical Micro-CT 2D images of the BBSs after (**A**) 28 days, (**B**) 90 days and (**C**) 180 days implantation in rabbit pulmonary arteries.

The 2D images of the typical explanted BBS segments (φ 5.0 × 8 mm) after 28, 90 and 180 days implantation in the rabbit pulmonary arteries were obtained by using Micro-CT to inspect corrosion extent (Figure 6). The BBS degraded very slightly after 28 days implantation and even kept its integrity after 6 months implantation. No strut fracture was observed at 28 and 90 days after implantation, and obvious degradation in a few struts at 180 days after implantation was observed (red arrows). The degradation products of the BBS scattered around the struts presented an indistinct appearance in the tissue. According to the *in vitro* mass loss test, the mass losses of the BBSs are 5.36 ± 1.81 wt%, 12.41 ± 2.17 wt% and 20.97 ± 2.64 wt% at 28, 90 and 180 days, respectively.

**Figure 7.**
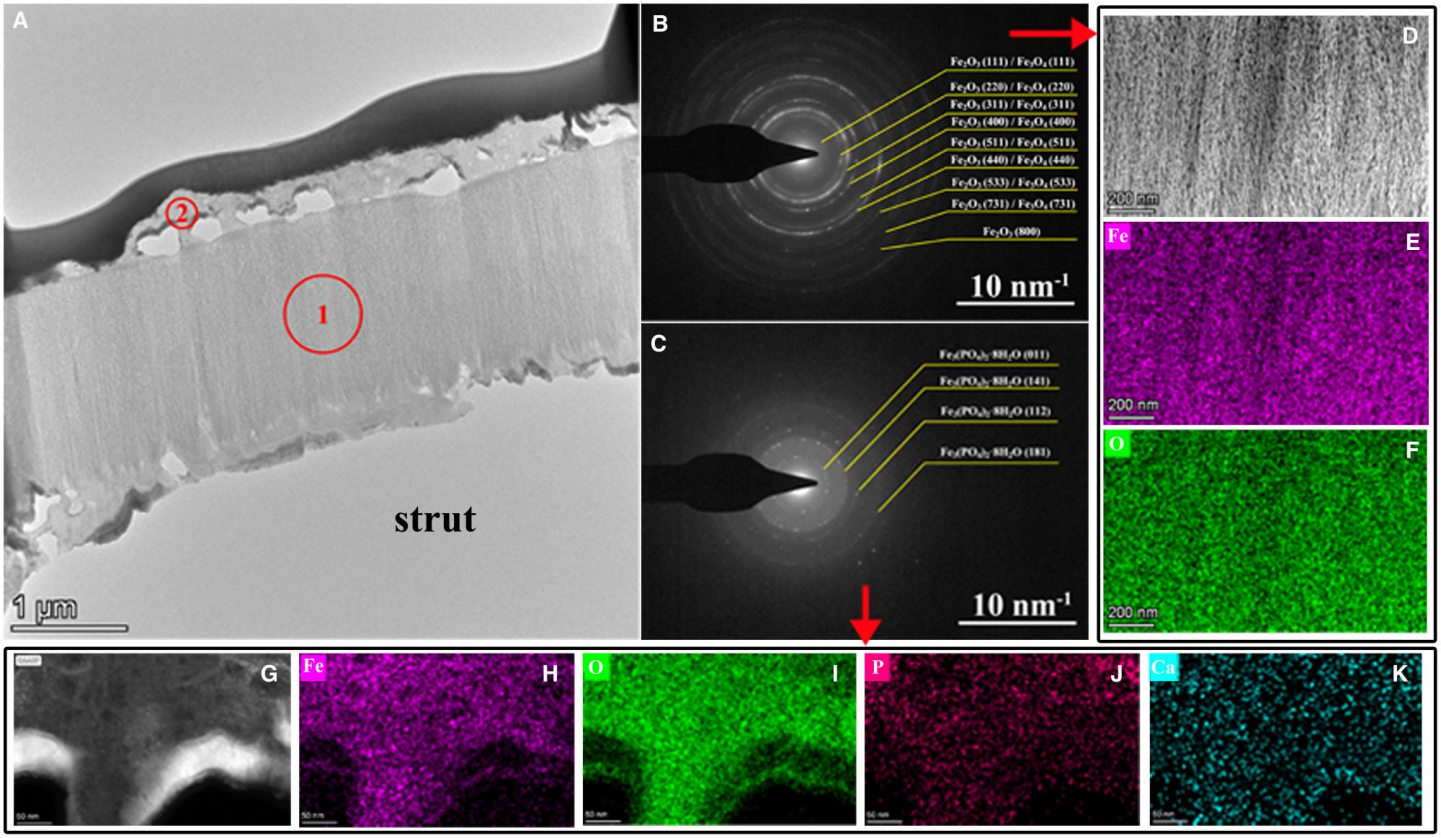
(**A**) HAADF-STEM image of the degradation products of the BBS after 6M implantation. (**B**) SAED pattern of the area 1 shown in (**A**). (**C**) SAED pattern of the area 2 shown in (**A**). (**D**) High-magnification HAADF-STEM image of the area 1 shown in (**A**). (**E**) and (**F**) EDS mapping of the area shown in (**D**). (**G**) High-magnification HAADF-STEM image of the area 2 shown in (**A**). (**H**–**K**) EDS mapping of the area shown in (**G**).

**Figure 8.**
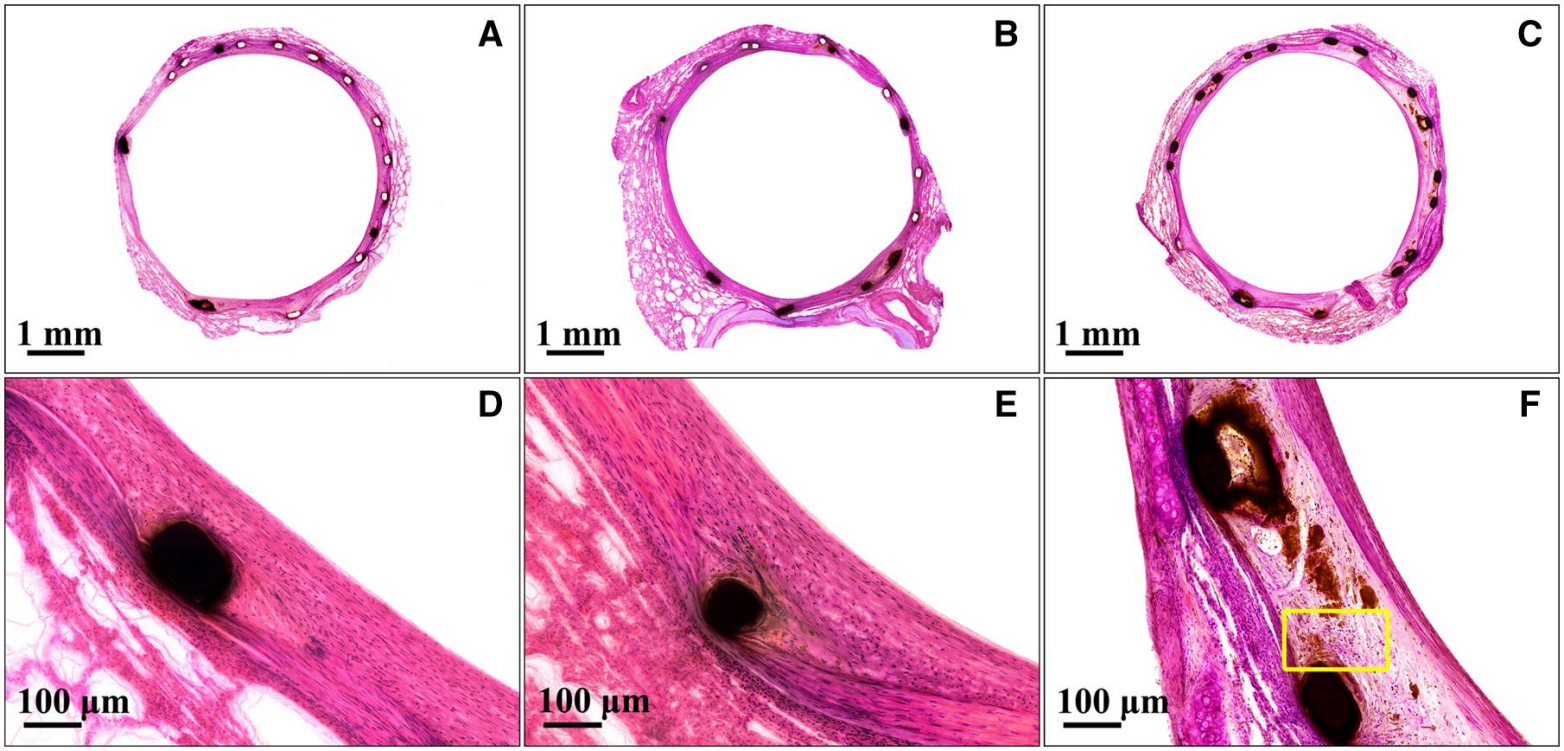
Typical histopathology images of the BBSs after (**A**) 28 days, (**B**) 90 days and (**C**) 180 days implantation in rabbit pulmonary arteries. (**D**–**F**) High-magnification images of (**A**–**C**).

STEM is used to characterize the phases and composition of degradation products of the BBS. Figure 7A is a typical bright field TEM image of the *in vivo* sample. It should be noted that the dark layer on top is a Pt film coated during FIB cutting. It is obvious that the core area (middle layer) of the sample is dense, while the edges are porous. A series of diffraction patterns (Figure 7B and C) were taken from varied areas indicated in Figure 8A to analyze the phase, and EDS mappings were used to understand the according chemical compositions (Figure 7D–K). Combining diffraction patterns and EDS mappings, it is clear that the composition of area 1 is Fe_2_O_3_ (JCPDS# no. 39-1346) and/or Fe_3_O_4_ (JCPDS# no. 75-0033). The edge area (area 2) is mainly composed of Fe_3_(PO_4_)_2_·8H_2_O (JCPDS# no. 30-0662).

#### Local tissue response

Figure 8A and D shows that the BBSs were completely covered by the neointima at 28 days after implantation. No scaffold degradation, inflammatory cells or phagocytes around the scaffold struts were found. After implantation for 90 days, few scaffold struts have signs of degradation. Neutrophils and lymphocytes around the scaffold struts were not detected yet, but some phagocytes around the scaffold struts were observed (Figure 8B and E). After implantation for 180 days, it can be seen that a few scaffold obviously degraded. More scaffold degradation products around the struts and obvious macrophages (yellow box) were observed, as shown in Figure 8C and F. No inflammatory reaction, malposition, thrombus, dissection or tissue necrosis was observed at every follow-up time point. The percentages of area stenosis of the BBS calculated from the histopathology images are approximately 18.1 ± 8.6%, 20.2 ± 5.9% and 20.4 ± 6.1% at 28, 90 and 180 days, respectively.

### First-in man implantation

**Figure 9.**
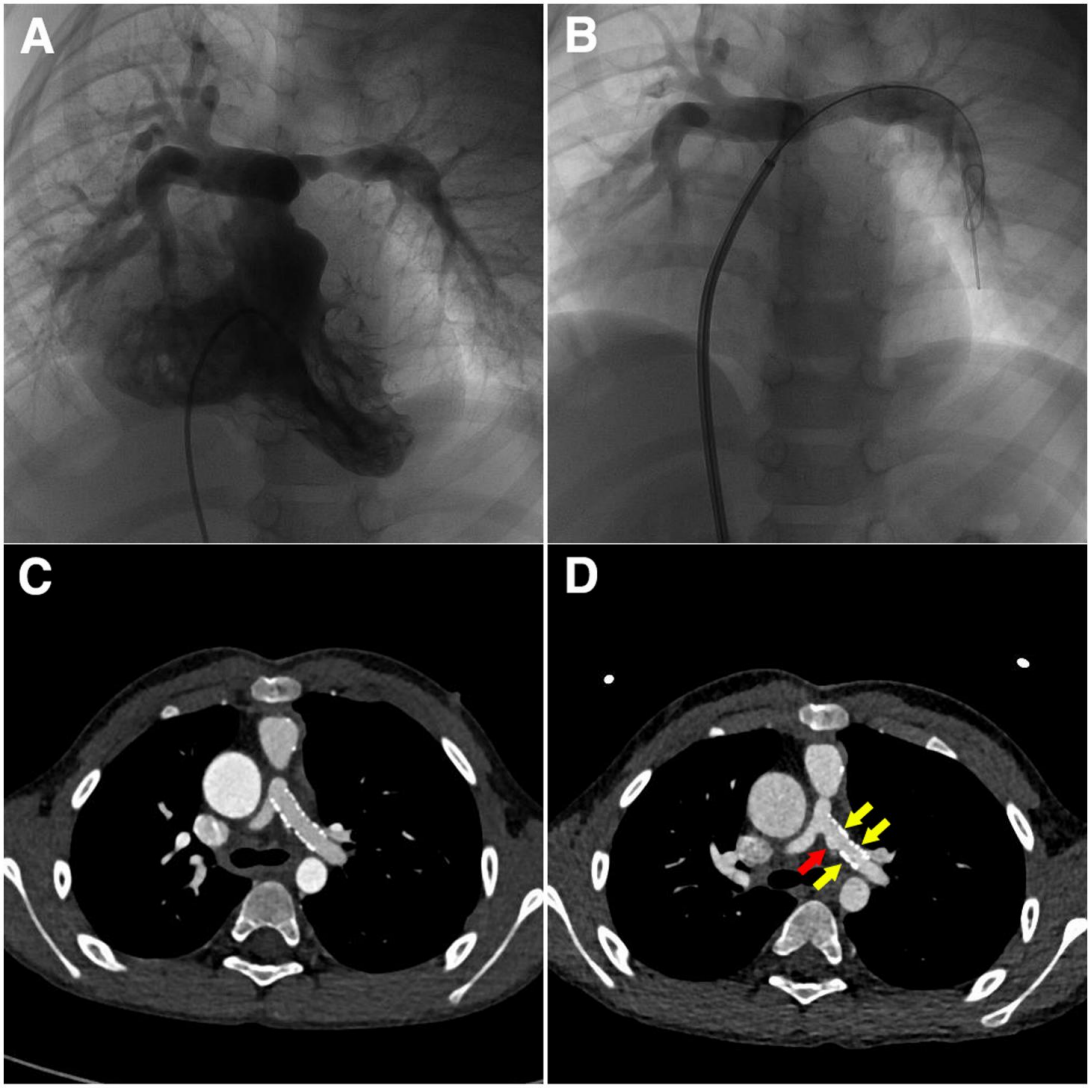
First-in-human implantation of the BBS. (**A**) DSA image before the procedure. (**B**) DSA image after the BBS implantation. (**C**) CT image at 1-year follow-up. (**D**) CT image at 2-year follow-up.

Figure 9 shows the results of the First-in-human implantation of the BBS. The patient, a 55-month-old child, had left pulmonary stenosis with ∼ 50% stenosis rate in diameter before the implantation (Figure 9A). A φ 8 × 23 mm BBS was implanted in the left pulmonary artery of the patient, and the corresponding angiographic image showed the open left pulmonary artery after implantation (Figure 9B). The diameter of the left stenosis pulmonary artery increased from 4.0 mm to 7.0 mm, and the pressure gradient between the main pulmonary artery and the left pulmonary artery decreased from 65 mmHg to 30 mmHg. As shown in Figure 9B, the BBS was well expanded and apposed after implantation. Serial observation through Spiral CT at 1- and 2-year follow-up were performed (Figure 9C and D). At 1-year follow-up, the scaffold still kept its integral, without apparent recoil, foreshorten or protruding strut. No scaffold thrombus or the other safety issues were observed. At time of the 2-year follow-up, the scaffold showed nearly the same results as 1-year follow-up except that part of the scaffold struts became wide (yellow arrows in Figure 9C), and the other part of the scaffold struts had not been identified (red arrow in Figure 9D) under CT examination due to the degradation and bio-absorption of the scaffold. No apparent lumen area loss was found at 1- and 2-year follow-up. The First-in-man implantation results show a good safety and effective profile, as well as an appropriate degradation profile of the BBS for children.

## Discussion

### Device performance of the BBS

**Figure 10.**
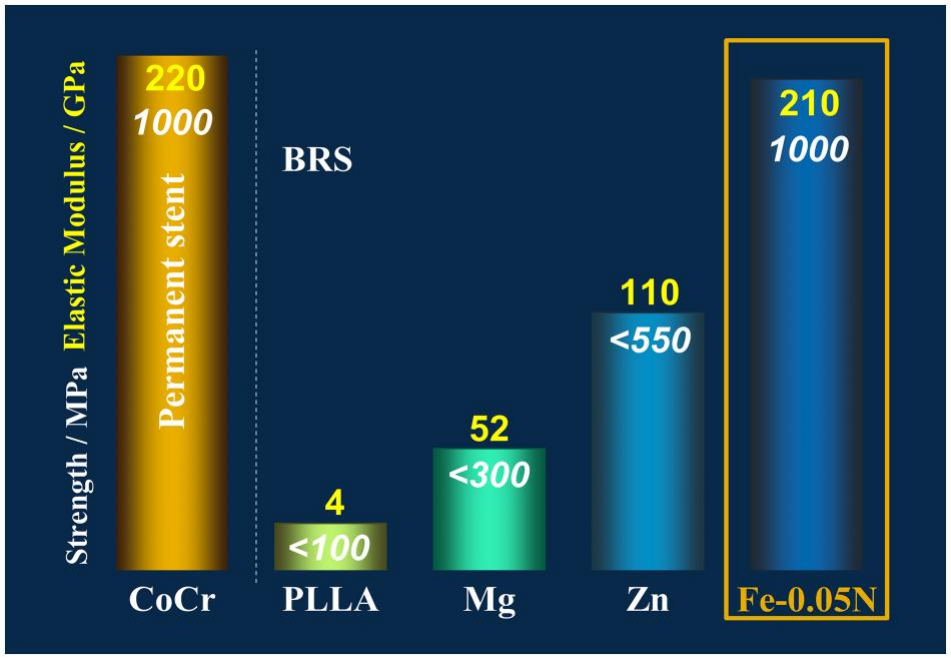
The mechanical performance of CoCr, Mg, Zn, Fe alloys and PLLA.

According to Zheng’s study results [15], metallic elements Ca, Mg, Zn and Fe are the potential candidates for matrix materials of the bioresorbable metals, and the nonmetallic elements O, C, N, etc., are potential candidates for alloying elements. Based on the currently reported types of bioresorbable scaffolds, the matrixes of bioresorbable scaffolds are polymer (PLLA), Mg, Zn and Fe alloys. Though the effort has been made to improve the mechanical properties of these materials, due to the limitations of their intrinsic properties, only the elastic modulus and tensile strength of the nitrided iron are similar to the CoCr alloy, which is the majority material for the mainstream permanent stent, and much higher than those of PLLA, Mg and Zn alloys [16–18], as shown in Figure 10. The high-strength nitrided iron was obtained by introducing N to pure iron matrix to form a dispersed second phase, Fe_4_N (as shown in Figures 2 and 3), which can also accelerate the corrosion of the iron matrix since the formation of galvanic corrosion between the second phase and the pure iron matrix [19]. However, if too much N is introduced into the iron matrix and resulting the coarsening of precipitated Fe_4_N grains, the plastic deformation ability of matrix will decrease, increasing the risk of scaffold fracture. Therefore, it is necessary to balance the tensile strength and plasticity of the material to fabricate scaffold with ideal device performance. In this study, results showed that the tensile strength of the nitrided iron tube increase with the N concentration, the tensile strength enters the platform period when the N concentration reaches 700 ppm, as shown in Figure 3. Combined with the clinical requirements of scaffold, which has high radial strength with the other acceptable mechanical performance, the preferred N concentration ranges from 700 to 1000 ppm in this work.

The elastic modulus is an inherent property that mainly depends on the material composition and is not sensitive to microstructure changes. The scaffold radial strength will be high when the elastic modulus and tensile strength are high under the same scaffold design. As an implantable medical device, the scaffold was crimped on the balloon in advance and then deployed in the lesion. The scaffold must be able to afford the deformation process, which requires good plastic deformation ability, especially for large-diameter scaffolds. The plastic deformation ability of the material mainly depends on the lattice structure of the material. The more slip systems, the stronger the plastic deformation ability. Iron alloy has a body-centered cubic structure with 12 slip systems, while magnesium and zinc alloy have a close-packed hexagonal structure with less slip systems [20], indicating the plastic deformation ability of iron alloy is generally better than that of magnesium and zinc alloys. Therefore, the elastic modulus, tensile strength and plastic deformation ability of the nitride iron are prior to the PLLA, Mg and Zn alloys [16–18], indicating the nitrided iron material is the most attractive material for manufacturing bioresorbable scaffold with high radial strength and good deformation ability, especially for the large-diameter scaffold.

For treating CHD children with pulmonary artery stenting, the stent diameter usually needs to be no less than 5 mm [21]. The scaffolds commonly used in clinical are with a diameter of 8 mm. As listed in Table 1, the wall thickness of the BBS with 8 mm diameter is only 105 μm with an 10.6% surface area @ NP, which is similar to that of a coronary CoCr stent (Xience Xpedition stent, ∼96 μm) [22], and is much less than both the commercial polymer bioresorbable coronary scaffold (NeoVas scaffold, ∼170 μm) [23] and metallic bioresorbable coronary scaffold (Magmaris scaffold, ∼164 μm) [24]. While the radial strength of BBS is up to 100 ± 5 kPa, enough to support the pulmonary artery. The minimum sheath for delivering the BBS with a diameter of 8 mm is 6F, which can be delivered inside the body of children above 1 year old. The recoil and foreshortening are small enough to benefit the precise deployment of the BBS. The maximal expansion diameter and side-branch diameter are big enough to satisfy post-dilation and side-branch patency. Sometimes, the scaffold or a certain scaffold unit has to be expanded to its design limit to ensure a good scaffold adhesion to the wall in large taper vessels and side-branch accessability. In such cases, the potential risk of scaffold fracture and subsequent in-scaffold restenosis is significant with severe deformation [25]. Therefore, in terms of scaffold design, the strut thickness, crossing profile, recoil and foreshortening of the scaffold should be as small as possible, while the radial strength, maximal expansion diameter and side-branch accessability of the scaffold should be as high as possible. However, the flexibility of the scaffold system is sacrificed if the radial strength and stiffness are too high. Thus, it is necessary to balance the device parameters to design the ideal scaffold with a thin strut, low profile, high flexibility, good radiopacity, sufficient radial strength, high over-expansion diameter, minimal recoil and foreshortening [26]. As values in Table 1 indicated, that the BBS has good comprehensive device performances. All BBSs were successfully implanted into the pulmonary arteries of the patient and clearly observed under pulmonary angiography. The BBS maintains a good vascular patency for all pulmonary artery stenosis.

### Effective and safety of the BBS

#### In vivo degradation of the BBS

In the present work, the BBS shows a featured non-uniform degradation by micro-CT images, which is consistent with *in vitro* study results [27]. According to our previous study [19], i*n vivo* degradation causes not only mass loss of the scaffold but also a decrease in radial strength. However, the degradation scaffold in the tissue can also provide enough radial strength since the tissue may act as a connector for the fractured or disintegrated scaffold struts to maintain structural integrity when the scaffold degradation is mild. The research results showed a nearly linear relationship between the radial strength of the degradation scaffold in the tissue and the mass loss, as well as the mass loss and the implantation time, especially for the first 12M after scaffold implantation. Afterwards, the degradation rate gradually slowed down and the whole degradation time of nitrided iron scaffold is about 3 years and the whole bioabsorption time is 7 years [28]. Since the BBS and the reported nitrided iron scaffold were both made from the nitrided iron tube and have a similar scaffold pattern design, it is reasonable that the degradation profile of the BBS is similar to the other nitrided iron scaffold in Lin et al. [19]. The mass loss of the BBS is slightly less than that of other nitrided iron scaffolds at 28, 90 and 180 days after implantation, which may be resulted from the large sizes of BBS used for animal study (Supporting Information Figure S2). The mass loss of the BBSs in rabbit pulmonary arteries also showed a nearly linear relationship with the implantation time. It can be inferred that the degradation rate of the BBS in pulmonary is ∼40 wt% @ 12 M after implantation, and the corresponding radial strength is ∼45 kPa; and the whole degradation time of the BBS is about 5–7 year since the size of the BBS is larger than the iron bioresorbable coronary scaffold. It is reported that the minimum radial strength of a stent should be no less than 24 kPa or 40 kPa with a safety factor [29], suggesting that the BBS can provide at least 1 year enough radial strength for the pulmonary artery. It is an ideal degradation time for CHD patients with PA stenting since the major issue associated with PA stenting in young children is that the lesions of most patients are usually tight scar tissues resulting from the surgical repair surgery, which may not grow as fast as the normal vessel and even may occur elastic recoil after balloon dilation, that why the young children are the need for stent re-dilatation [30, 31] and the success rate of the pulmonary artery balloon angioplasty is low. Therefore, the ideal bioabsorbable pulmonary artery scaffold for palliative treatment should provide enough radial strength until the next re-intervention or repair surgery.

Meanwhile, the scaffold does not restrict the pulmonary artery. We also observed that the iron bioresorbable coronary scaffold did not restrict the vessels and grew with the vessels in porcine coronary arteries at 17 M after implantation (the segments in the yellow boxes and between the yellow lines, as shown in Supporting Information Figure S1). If there is a need for blood vessels to grow, the scaffold will not constrain the blood vessel growth. However, the nonuniform degradation may cage the vessel around the undegraded scaffold segments (the parts between red lines shown in Supporting Information Figure S1B), which is a disadvantage for children with a requirement of growth. Therefore, we designed a scaffold strut with the breakpoints (Figure 1) to avoid the above problem. These microholes on straight strut segments may control the scaffold-dismantling time point to get rid of scaffold restriction to vessel growing. It is a favorite design of big-size scaffolds since the design of breakpoints on the struts can reduce the iron amount in the BBS without reducing its mechanical performance.

#### Biocompatibility of the BBS

It was reported that stent strut thickness is one of the critical factors for endothelial coverage [32]. The thin stent strut thickness has lower disturbance on the blood flow and greater endothelial coverage [33], contributing to a lower risk of thrombosis [34]. In this study, the strut thickness of the BBS is only ∼105 μm, which is thinner than the commercial off-label-use stent for PA stenting [35]. The animal results showed a complete endothelialization of the BBS after 28 days of implantation in the rabbit pulmonary artery, indicating low or no cytotoxicity for the nitrided iron material. The endothelialization research results showed the BBS has fast endothelialization velocity, meaning low *in vivo* thrombosis risk.

The composition and distributions of degradation products of the BBS in rabbit pulmonary artery were characterized as Fe_2_O_3_, Fe_3_O_4_ and Fe_3_(PO_4_)_2_, which is the same as thermodynamic calculation results, as shown in Figure 7. In this study, the elements P and Ca were also detected in the outermost layer of the degradation products. Lin et al. also reported that a Ca/P layer formed at the outer layer of the degradation products of a nitrided iron scaffold in the rabbit abdominal aorta [19]. A Ca/P area around the degradation products of the PLLA scaffold, Mg alloy scaffold and Zn alloy scaffold was also observed in animal studies [36–38]. An issue of concern is that Ca/P or passive layer may prevent the dispersion of the degradation products and then prevent the degradation of the residual iron struts. However, a tendency of dispersion of degradation products was observed in this work, which is realized through macrophage engulfing the degradation products and moving to the adventitia, as shown in Figure 8, indicating the phosphate calcium layer is not dense or discontinuous, which may be caused by serum proteins [39], as shown in Figure 7. The cells can pass through this layer to clear up the degradation products and repair tissue. Tissue regeneration within the original strut has also been found in porcine coronary artery implanted with nitrided iron scaffold [28]. The bioresorption mechanism of the nitrided scaffold has been studied systematically in our previous study [19, 28]. The solid degradation products can be engulfed by macrophages from *in situ* to the adventitia, enter the lymphatics, and finally, travel to lymph nodes, similar to the absorption mechanism of internal bleeding in the human body. The degradation products of the BBS probably have the same transfer way since the degradation products can also be engulfed by macrophages and move to the adventitia as there are abundant lymphatics and lymph nodes in the lung [40]. The 2-year follow-up of the First-In-Man clinical trial has demonstrated that part of the scaffold wasn’t identified under the CT image, no calcification was observed, indicating the degradation products of the BBS can be bio-absorbed in the human pulmonary artery, which will not cause the formation of calcification, as shown in Figure 9. The reason that the macrophages entering the lymphatics were not observed in this study may be due to the short follow-up time. In this case, a long-term animal study is placed and is currently ongoing.

The amount of iron that enters and leaves the body each day in a healthy person is 35–45 mg/kg and 1–2 mg of iron [41], suggesting that the risk of systemic toxicity of the BBS is probably very low since the mass of the BBS (φ 8 × 23 mm) is approximately 55 mg and has a long degradation period. Our previous study showed that no abnormalities were observed in gross observation and histopathologic analysis of the heart, liver, spleen, liver and lung of mini-pig implanted with nitrided iron scaffold at 53 months follow up [19]. No abnormalities were observed in the histopathologic analysis of scaffolded segments up to 7 years [28], indicating that the degradation products of nitrided iron scaffold that had existed *in vivo* for a very long time were safe. In addition, no iron overload has been detected in clinical trials up to now, which might support the above speculation. Our future work will focus on reducing the amount of iron and insoluble degradation products by further improving the tensile strength of the nitrided iron tube and adding iron chelator to form soluble substances.

### Safety, efficacy and clinical prospects of the BBS

Usually, the origin of pediatric devices is based on the maturity of devices for adult patient. The development of bioresorbable device for pediatric cardiology is in the same situation. Few bioresorbable device for children with CHD was reported [42]. Therefore, developing bioresorbable device for children is a meaningful and attractive matter, which could potentially improve the strategy for the therapy of children with CHD and solve long term problems associated with the use of permanent device for children with CHD. The BBS is designed specifically for children with CHD in view of the growth characteristics, and it had also given a choice to those children with complex CHD who currently do not have good treatment options.

This is the first long term report of implantation of iron bioresorbable scaffold into vein (the pulmonary artery is actually vein). It is known that, most arterial vascular stenosis is caused by atherosclerosis, which is quite different from venous vascular stenosis, especially scar tissue of pulmonary artery after surgical repair. Therefore, the effects of their interventional treatments have remarkable difference. The atherosclerotic plaque can be pressed by the balloon dilation without excessively damaging the smooth muscle, the immediate success of balloon dilation is acceptable. However, venous vascular stenosis caused by proliferation formed after thrombus intensification or scar tissue are elastic, and the scar tissue is mostly fibrous connective tissue with strong binding force. Therefore, the immediate success of balloon dilation is low, which determines that stenting is the most appropriate treatment for the venous vascular stenosis. In addition, the blood flow velocity of artery is faster than vein, meaning low thrombosis risk in artery, which requires rapid endothelialization of scaffold in vein. In this research, both animal study and clinical outcomes showed that BBS has a rapid endothelialization and can provide enough radial strength, no thrombus was observed [43]. Recently, the follow-up of the BBS implanted in right ventricular outflow tract (compassionate use in the United States) were reported [44], the follow-up results meet the clinical requirements. These clinical results showed that the BBS is a promising bioresorbable scaffold that can be safely and effectively applied to children with CHD.

Children with other indications of congenital heart disease, such as aortic stenosis (AS) and pulmonary valve stenosis (PVS), also urgently require suitable devices. Covered stent and pulmonary valve stent maybe the suitable devices for AS and PVS treatment. However, the stents used to treat these two diseases are permanent stents [45, 46], which is incompatible with the growth needs of children. The new structure iron bioresorbable scaffold has been designed and developed based on the BBS for children with AS and PVS indications of congenital heart disease. A covered BBS was prepared, to implant in AS of a child with CHD. Up to now, the follow-up results showed the covered scaffold is safe and effective. Furthermore, if the iron bioresorbable scaffold is implanted to a young child, then the child may need to implant 1 or 2 more iron bioresorbable scaffold to keep the effective lumens until adulthood. Our previous animal study has demonstrated that the repeated implantation of iron bioresorbable scaffolds is feasible [47].

### Study limitations

The present clinical outcomes in this work could only provide a preliminary evaluation of the effectiveness and safety of the BBS due to the relatively small number of enrolled patients currently. In addition, long-term clinical results are pending for the effectiveness and safety of the BBS, as well as the change in degradation and impact on the pulmonary artery wall, which is different from the arterial blood vessel, over an extended period.

## Conclusions

In this study, the BBS is made of high-strength nitrided iron tube, and shows excellent device performances. Due to its thin wall thickness and good mechanical properties, the BBS can provide enough radial strength for PAS. The *in vivo* degradation profile of the BBS used for pulmonary artery scaffold shows a reasonable degradation rate. The degraded BBS provides radial strength and does not restrict the pulmonary artery. The degradation products of the BBS in rabbit pulmonary artery are characterized as Fe_3_O_4_ near the remaining iron struts, Fe_2_O_3_ in the middle area, Fe_3_(PO_4_)_2_ in the outmost area, which can be cleared away by macrophages from *in situ* to adventitia and probably enter the lymphatics and finally travel to lymph nodes. The complete endothelialization of the BBS in the pulmonary artery after 1 month of implantation is observed, and no scaffold thrombus, necrosis or serious inflammatory responses are found. The clinical outcomes show the device success rate is 100%, and no severe safety issues are observed. Therefore, this study demonstrates that the BBS is a promising therapy for CHD patients with PAS, which may bring big benefits to the patients, especially for newborns and infants.

## Supplementary Material

rbaf041_Supplementary_Data
