# Supplementary material for: A novel iron bioresorbable scaffold: a potential strategy for pulmonary artery stenosis
Source: Regen Biomater. 2025 May 13;12:rbaf041. doi: 10.1093/rb/rbaf041 (PMC12202099; doi:10.1093/rb/rbaf041)
Supplement: rbaf041_Supplementary_Data [file rbaf041_supplementary_data.zip › Supplementary Materials.docx]

**Supplementary Materials**

**
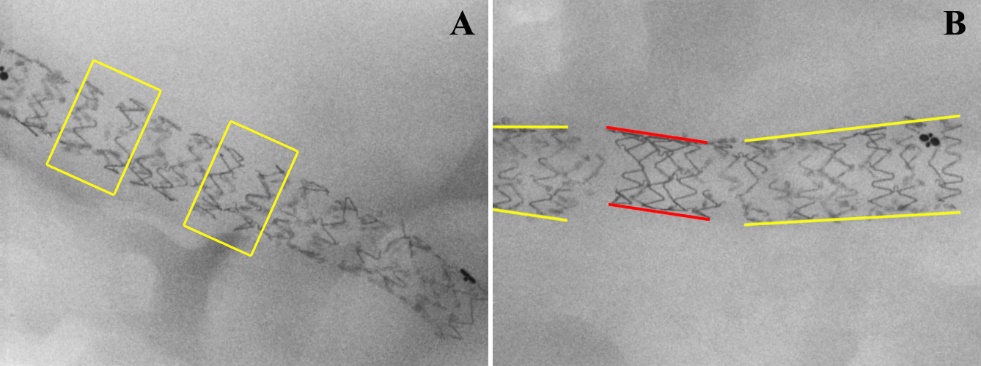
**

**Fig.S1.** Micro-CT images of the iron bioresorbable coronary scaffold in porcine coronary artery at 17 M after implantation. (A) The scaffold becomes longer after the scaffold degradation (yellow boxes). (B) The diameter of the scaffold becomes larger after the scaffold degradation (the segments between yellow lines).

**

**

**Fig. S2.** The mass loss of the BBS and the other nitrided iron scaffolds in rabbit model, respectively.
